# Therapeutic isolation in psychiatry

**DOI:** 10.1192/j.eurpsy.2021.1338

**Published:** 2021-08-13

**Authors:** H. El Kefi, W. Kabtni, S. Boughalmi, I. Gafsi, A. Baatout, W. Krir, A. Oumaya

**Affiliations:** 1 Psychiatric Unit, Military Hospital of Tunis, tunis, Tunisia; 2 Psychiatry, military medical school, tunis, Tunisia

**Keywords:** patient isolation, administration and organization

## Abstract

**Introduction:**

Therapeutic seclusion, consist of locking a patient alone in a room dedicated to this type of care. It poses real questions about respect for fundamental rights, in particular the right to liberty.

**Objectives:**

The objectives of our work were to evaluate the practices of psychiatric doctors and nurses in seclusion.

**Methods:**

Our study is descriptive-based. Using a self-questionnaire, a set of questions about the decision of seclusion, its prescription, its means, its surveillance, the information of the patient and his relatives as well as the characteristic of the seclusion room, were asked. Our target population consisted of medical and paramedical staff working in the HMPIT Psychiatry Department.

**Results:**

Our study covered eleven doctors and fourteen nurses. The average age was 35 years with 52% having more than 5 years of psychiatric experience. Fifty-two percent used seclusion while 72% had no specific training. Twelve (12%) percent felt that no accidents could occur in seclusion. Twenty percent (20%) did not find it necessary to transcribe the monitoring parameters on the medical file.

**Conclusions:**

Our study showed that the psychiatric staff lack sufficient exposure to in the area of therapeutic seclusion. A seclusion protocol has been drawn up to guarantee patient safety.

